# Unusual Obstruction of Nasojejunal Feeding Tube

**DOI:** 10.4103/1319-3767.56092

**Published:** 2009-10

**Authors:** Mohan Gurjar, Bhaskar P. Rao, Afzal Azim

**Affiliations:** Department of Critical Care Medicine, Sanjay Gandhi Postgraduate Institute of Medical Sciences, Lucknow, UP, India

**Figure 1:**
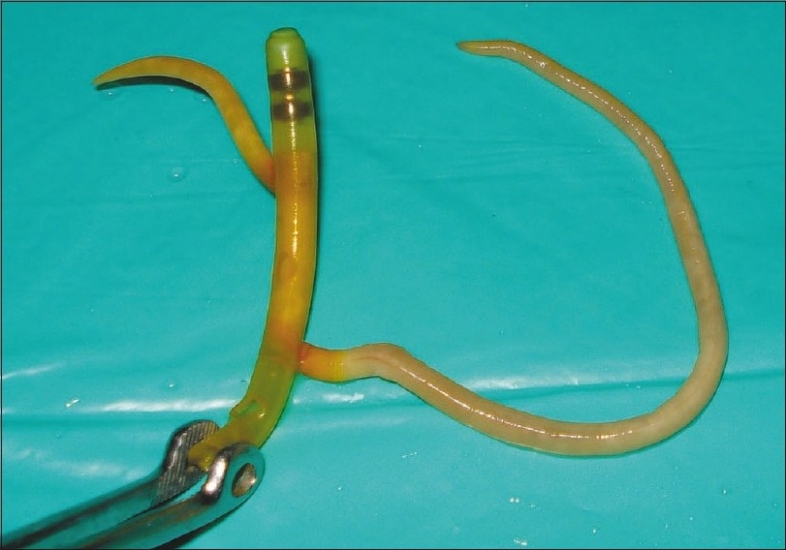
Feeding tube occlusion by *Ascaris lumbricoides*

Despite good practice of caring, blockage of feeding tube is a common problem. We encountered a patient with severe acute pancreatitis in the ICU with a nasojejunal tube for enteral feeding. Later on, due to obstruction in the feeding tube, we took it out and found, unexpectedly, the lumen of the feeding tube occluded by *Ascaris lumbricoides* [Figure 1].[1,2] Therefore, this occlusion by *A. lumbricoides* could be considered as a cause of feeding tube obstruction, especially in endemic areas where the burden of this disease is huge.[3]
